# Evaluation of hemodynamic characteristics of iliac vein in chronic venous disease and iliac vein compression syndrome patients using magnetic resonance image: A prospective study

**DOI:** 10.1016/j.jvsv.2025.102247

**Published:** 2025-04-08

**Authors:** Tianchen Xie, Xingyu Su, Yan Shan, Min Zhou, Yong Ding, Xu Li, Zhenyu Zhou, Sheng Fang, Chenghao Yang, Zhenyu Shi

**Affiliations:** a Department of Vascular Surgery, Zhongshan Hospital, Fudan University, Shanghai, China; b School of Medical Technology, Beijing Institute of Technology, Beijing, China; c Department of Radiology, Zhongshan Hospital, Fudan University, Shanghai, China

**Keywords:** Chronic venous disease, Hemodynamics, Magnetic resonance image, May-Thurner syndrome

## Abstract

**Objective:**

The purpose of this study was to explore the characteristics of hemodynamic parameters of iliac vein of patients with chronic venous disease (CVD) using two-dimensional (2D)-phase contrasted (PC) and four-dimensional (4D)-Flow magnetic resonance imaging (MRI) and to test the diagnostic and grading efficiency of 2D-PC and 4D-Flow MRI in CVD and iliac vein compression syndrome (IVCS).

**Methods:**

Consecutive patients with CVD diagnosed in the outpatient department from 2023 to 2024 were enrolled in this study. Demographic data and medical records of the patients were also collected. The CEAP classification, Villalta score, and Venous Clinical Severity Score (VCSS) were used to evaluate the severity of lower limb symptoms. After computational tomography venography (CTV) scans to verify iliac vein compression, every patient underwent 2D-PC and 4D-Flow MRI scanning. Circle CVI42 software was used to perform data post processing. The inferior vena cava (IVC), common iliac vein (CIV), and common femoral vein (CFV) were chosen to acquire hemodynamic parameters by MRI. The hemodynamic parameter included flow rate (FR) per cardiac cycle, FR per minute, peak flow velocity (FV) and minimum FV measured by 2D-PC MRI and FR per cardiac cycle, peak FV and pressure gradient measured by 4D-Flow MRI. We analyzed the consistency of hemodynamic parameters between 2D-PC and 4D-Flow MRI, the differences in hemodynamic parameters between symptomatic and asymptomatic limbs and limbs with and without iliac vein compression, and the correlation between parameters and severity of symptoms.

**Results:**

A total of 34 individuals, including three healthy volunteers, 15 patients with CVD and iliac vein compression, and 16 patients with CVD without IVCS, were enrolled in this study. Hemodynamic parameters measured by 2D-PC and 4D-Flow MRI complied with the flow rate conservation and maintained consistency (*P* < .01). There was a statistically significant difference in the FR of the CIV and FR difference between the CIV and CFV measured by 2D-PC and 4D-Flow MRI between symptomatic and asymptomatic limbs (2D-PC MRI: FR of CIV: 6.0 ± 3.1 vs 8.5 ± 5.1; *P* = .01; FR difference: 1.6 ± 2.1 vs 3.6 ± 4.3; *P* = .01) (4D-Flow MRI: FR of CIV: 6.9 ± 2.8 vs 8.7 ± 4.2; *P* = .04; FR difference: 3.0 ± 2.8 vs 4.8 ± 3.5; *P* = .05), and limbs with and without iliac vein compression (2D-PC MRI: FR of CIV: 5.3 ± 3.0 vs 7.6 ± 4.4; *P* = .03; FR difference: 1.3 ± 2.7 vs 2.8 ± 3.4; *P* = .04) (4D-Flow MRI FR of CIV: 6.1 ± 2.6 vs 8.2 ± 3.7; *P* < .01; FR difference: 2.1 ± 3.7 vs 4.6 ± 3.1; *P* = .04). The FR of the CIV and the FR difference between the CIV and CFV were negatively correlated with symptom severity in all affected limbs (2D-PC MRI: FR of CIV: *P* < .01; r = −0.3; FR difference: *P* = .03; r = −0.3). There was a potential negative correlation between the FR of the CIV in limbs with iliac vein compression and the severity of symptoms (2D-PC MRI: FR of CIV: *P* = .07; r = −0.4).

**Conclusions:**

In conclusion, hemodynamic parameters provided by 2D-PC and 4D-Flow MRI possess the potential clinical value of evaluating CVD and iliac vein compression.

**Article Highlights**

• **Type of Research:** A single-center, prospective, non-randomized cohort study
• **Key Findings:** In 31 patients with chronic venous disease and three healthy volunteers, two-dimensional (2D)-phase contrasted (PC) and four-dimensional (4D)-Flow magnetic resonance imaging (MRI) were used to acquire the hemodynamic parameters of the inferior vena cava, common iliac vein (CIV) and common femeral vein. There was consistence existing between the hemodynamic parameters measured by 2D-PC and 4D-Flow MRI. In addition, the flow rate-related parameters of CIV were significantly different between symptomatic and asymptomatic limbs, which was illustrated between limb with and without iliac vein compression as well. Furthermore, the flow rate-related parameters of the CIV correlated with the severity of symptoms in all limbs and limbs with iliac vein compression.
• **Take Home Message:** The decreased iliac vein flow measured by MRI correlated with the severity of lower limb venous symptoms. 2D-PC and 4D-Flow MRI possess the potential clinical value of assisting chronic venous disease and iliac vein compression diagnosis and lower limb symptom classification.

Chronic venous disease (CVD) can cause a wide range of symptoms and signs, such as varicose veins, limb swelling, hyperpigmentation, venous claudication, and chronic venous leg ulcers, which have a high prevalence in the general adult population.^1^ Iliac vein compression syndrome (IVCS), also known as May-Thurner syndrome or Cockette’s syndrome, is thought as an important cause of CVD, in which the iliac vein is compressed by the iliac artery.^2^ However, plenty of patients with iliac vein compression are asymptomatic.^2^ Furthermore, there are many patients with CVD without iliac vein compression. A previous study illustrated that symptomatic iliac vein compression can reach 2% to 5% in people with CVD, and up to 18% to 49% in people with deep venous thrombosis.^3^ Some researchers have treated the presence of iliac vein compression as a normal anatomical variant in the asymptomatic population.^4^^,^^5^ Therefore, the severity of anatomical alterations may not be related to the venous symptoms of the lower limbs, which means that it is not accurate to diagnose IVCS according to morphologic features alone. Thus, hemodynamic parameters of lower limb veins have been tried to evaluate the severity of IVCS using ultrasound^6^ and conventional venography.^7^ However, previous studies have shown that these methods are neither sensitive nor precise for the evaluation of IVCS.^8^

Two-dimensional-phase contract magnetic resonance imaging (2D-PC MRI) has gradually become more common as a research tool to investigate anatomy, angiography, and flow and velocity information noninvasively.^9^ In recent years, 4D-Flow MRI has emerged as an in vivo flow imaging technique that enables visualization and quantification of complex flow patterns.^10^ Compared with 2D-PC MRI, 4D-Flow MRI can not only provide flow parameters such as flow velocity and flow rate, but also stress and characteristic parameters such as wall shear stress, change in pressure gradient, pulse wave velocity, and turbulent kinetic energy,^11^ which have been used in the heart, large vessel arteries, and portal and intracranial veins.^10^^,^^12^, ^13^, ^14^ However, few studies reported the application of 2D-PC MRI and 4D-Flow MRI in CVD or IVCS. Therefore, the purpose of this study was to explore the characteristics of the hemodynamic parameters of IVCS using 2D-PC MRI and 4D-Flow MRI, testifying to the diagnostic and grading efficiency of 2D-PC MRI and 4D-Flow MRI in CVD and IVCS.

## Methods

### Patients

This prospective, single-center cohort study was approved by the Institutional Review Board of Zhongshan Hospital, Fudan University (B2024-476). After providing written informed consent, patients with CVD diagnosed in the outpatient department underwent MRI examination. The exclusion criteria were: (1) pregnancy; (2) severe inferior vena cava (IVC) compression; (3) deep venous thrombosis and post-thrombotic syndrome; (4) patients with body mass index >30 kg/m^2^; and (5) claustrophobia and arrhythmia. Before the MRI examination, every patient was instructed to undergo IVC and iliac and femoral vein computed tomography venography (CTV) and lower limbs vein color Doppler ultrasound to verify whether the iliac vein was compressed and there was evident femoral vein reflux. The patients with multiple compression sites in ipsilateral iliac vein and femoral vein reflux persisting over 1 second were excluded as well. The anatomic characteristics, including the area stenosis of compression and confluence angle of the CIV, were recorded.^15^ The area stenosis of compression was defined as 1 − (lumen area at stenosis/distal normal lumen area) × 100%, which could be measured and calculated in a 3D model established by MIMICS 20.0 (Materialise) and Geomagic Studio 2012 (Geomagic, Inc) software referring to the CTV (Supplementary Fig 1 *A* and *B*, online only). According to the Guidelines for the Diagnosis and Treatment of Common Venous Diseases (2022 Edition),^16^ the criterion for iliac vein compression in CTV was that the area stenosis of the compressed CIV in the symptomatic limbs was greater than 50% compared with the area of the distal normal iliac vein. Furthermore, the confluence angle of the CIV was defined as the angle between the center line of the IVC and CIV, which could be measured and calculated in the coronal CTV image (Supplementary Fig 1, *C*, online only). Demographic data and medical records of the patients were also collected. The CEAP classification, Villalta score, and Venous Clinical Severity Score (VCSS) performed by surgeons were used to evaluate clinical status.

### MRI hemodynamic parameters acquisition protocol

2D PC-MRI and 4D-Flow MRI data were acquired for each subject using a 3.0 T Philips Ingenia MRI scanner, without the use of any contrast agent. According to the MRI scan protocol published in a previous study,^17^ to ensure comparability between 2D-PC MRI and 4D-Flow MRI measurements, all data acquisition was performed during the same examination session, and every subject stayed approximately 30 to 45 minutes for the overall scan. All subjects were examined in the supine position, headfirst, using ECG gating, with the abdominal coil placed on the lower abdomen. Before MRI scanning, all subjects were required to practice holding their breath and to ensure that they could comply with the instructions of the radiologist during the scanning period. All MRI scans were performed by a radiologist, and both the surgeon and radiologist were blinded to each other’s results.

**Fig 1:**
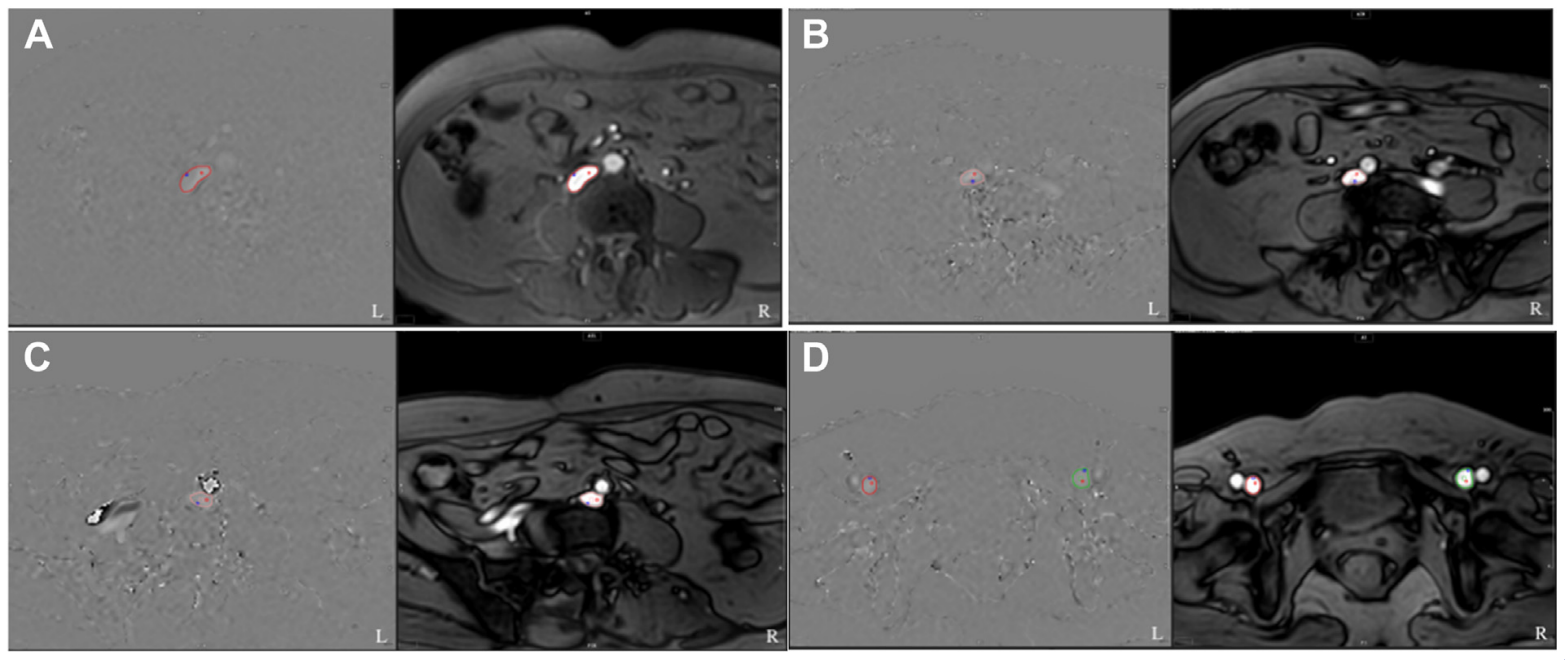
The regions of interest (ROIs) of the inferior vena cava (IVC) **(A)**, right common iliac vein (RCIV) **(B)**, left common iliac vein (LCIV) **(C)**, and bilateral common femoral vein (CFV) **(D)** in axial phase (L) and amplitude (R) image.

**2D-PC MRI.** The imaging sequence was a Q-flow sequence based on the phase contrast. The coronal image of the IVC T2WI was used as a reference, with the IVC proximal to the iliac vein bifurcation, CIV, and CFV used as the scanning planes. The scanning direction was perpendicular to the target blood vessels. If iliac vein compression was observed in the previous CTV image, the scanning plane of the CIV was normal CIV distal to the compression site, referring to the CTV scan. The breath-holding scanning method was used for ECG monitoring for heart rate detection. The two groups of cross-sectional images were the amplitude and phase, as shown in Fig 1. The scan parameter for 2D-PC MRI sequence were adjusted as follow: repetition time (TR) 4.8 ms, echo time (TE) 3.2 ms, pixel spacing 1.17 mm, slice thickness 8 mm, and the number of frames 30. Velocity encoding (VENC) was initially set to 50 cm/s and adapted until the image had no confusion, which was close to the real blood flow velocity.

**4D-Flow MRI**. A 2D-PC scan was performed at the target blood vessel to be measured, perpendicular to the inferior vena cava and iliofemoral vein. Then unidirectional velocity encoding was performed in the direction of the through-plane, and different VENC values were set to avoid phase wrapping. After selecting the best value, 4D-Flow MRI was performed. The scan parameters for 4D-Flow MRI sequence were as follow: TR 4.4 ms, TE 2.8 ms, flip angle 7°, slice thickness 2.5 mm, VENC 50 to 80 cm/s.

### Data post-processing

The original images of the Q-flow sequences were transmitted to Circle CVI42 (Circle Cardiovascular Imaging). Regions of interest (ROIs) were delineated along the contours of the IVC, CIV, and CFV according to the axial amplitude map (Fig 1). Hemodynamic parameters, including flow rate (FR) per cardiac cycle, FR per minute, peak flow velocity (FV), and minimum FV, were automatically calculated using the software.

**Fig 2:**
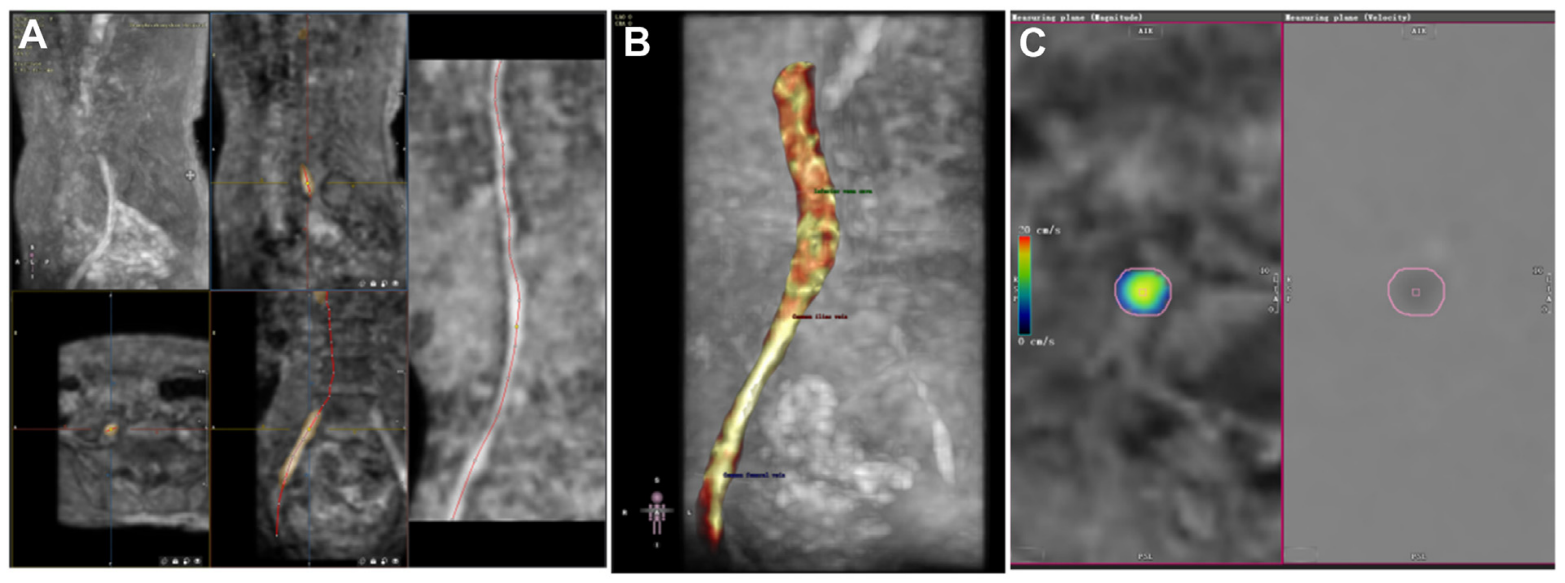
The data processing of four-dimensional flow magnetic resonance imaging (4D-Flow MRI), including determining the center line of lumen and adjusting the contours **(A)**, the plane of measurement **(B)**, and adjusting the region of interest (ROI) of measurement **(C)**.

**Fig 3:**
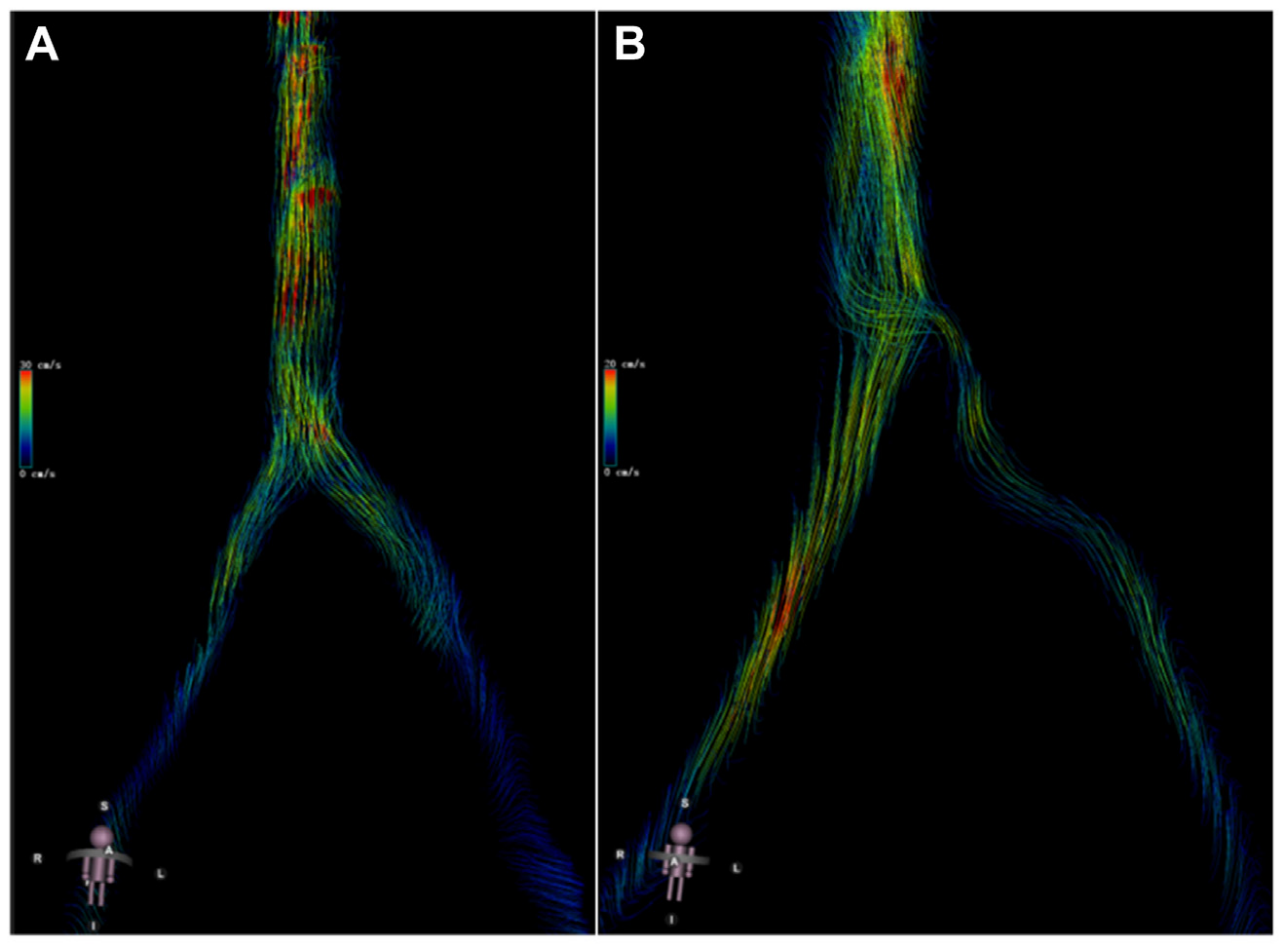
The streamline charts of the inferior vena cava (IVC) and common iliac vein (CIV) for two patients: Patient **A**, who has chronic venous disease (CVD) in the right lower limb, and Patient **B**, who has left CIV compression.

The original statistics of 4D-Flow MRI, including an amplitude image and a sequence of phases representing three directions, were transmitted to the Circle CVI42 (Circle Cardiovascular Imaging). ROIs of the IVC, CIV, and CFV were extracted, and blood flow information was automatically analyzed and visualized using software. Measure planes were placed vertically on the IVC, CIV, and CFV, and hemodynamic parameters of every plane were acquired and quantified, including the FR per cardiac cycle, peak FV, and pressure gradient. Similar to 2D-PC MRI, the measured plane of the CIV with iliac vein compression was the normal iliac vein distal to the compression site, referring to the CTV scan. The post-processing of the 4D-Flow MRI is shown in Fig 2. The streamlined charts of the symptomatic limb and limb with iliac vein compression are shown in Fig 3. The hemodynamic parameters including definitions and units measured by 2D-PC and 4D-Flow MRI are listed in Supplementary Table I (online only).

### Statistical analysis

Excel table (Microsoft Office 16.63) was used to record the demographic information, severity of CVD, and hemodynamic statistics of 2D-PC MRI and 4D-Flow MRI. IBM SPSS 23.0 was used to analyze the data. Continuous variable data that met the normal distribution were expressed as medians and standard deviations (SDs). The abnormally distributed continuous variable was represented by the median and quartile M (interquartile range [IQR]). The intraclass correlation coefficient (ICC) was calculated to evaluate the consistency of hemodynamic parameters between 2D-PC MRI and 4D-Flow MRI. The Student *t*-test and nonparametric tests were used to compare the differences in hemodynamic parameters acquired from 2D-PC MRI and 4D-Flow MRI between symptomatic and asymptomatic limbs and limbs with and without iliac vein compression. Statistical significance was set at *P* < .05. Spearman’s rank analysis was used to evaluate the correlation between hemodynamic parameters and CEAP classification of limbs, and Pearson’s analysis was used to evaluate the correlation between hemodynamic parameters and anatomic parameters, and Villalta and VCSS scores of the limbs. Statistical significance was set at *P* < .05, which indicated the correlation was considered significant, with .05 ≤ *P* < 1.00 considered potentially statistically correlated.

## Results

### Patient characteristics and severity of symptoms

There were 34 individuals (42 symptomatic limbs and 26 asymptomatic limbs) enrolled in this study, including three healthy volunteers, 15 patients with iliac vein compression, and 16 patients with other CVD. The patients with IVCS consisted of five with bilateral iliac vein compression (5 left and 5 right limbs) and 10 with unilateral iliac vein compression (10 left limbs). The patients with other CVD consisted of six with bilateral symptomatic (6 left and 6 right limbs) and 10 with unilateral symptomatic (8 left and 2 right limbs). In this study, there were no asymptomatic lower limbs with iliac vein compression. The mean patient age was 61 ± 13 years, the female/male ratio was 15:19, and the mean body mass index was 25.1 ± 3.5 kg/m^2^. Patients’ demographic information and characteristics are shown in Table I.

**Table I:** The patients’ demographic information and characteristics

Variable	Data
Female/male gender	15:19
Age, years	61 ± 13
Addicted to alcohol	3
History of smoking	0
Hypertension	12
Diabetes	1
Cardiovascular disease	4
Cerebrovascular disease	5
Patients with CVD with limbs with varicose veins	12
Patients with iliac vein compression with limbs with varicose veins	7
Median value of area stenosis	51.8% (49.6%-62.3%)
Median value of LCIV angle	45.5 ± 10.2°
Median value of RCIV angle	20.1 ± 5.7°

*CVD,* Chronic venous disease; *LCIV,* left common iliac vein; *RCIV,* right common iliac vein.

Data are presented as number, median (interquartile range), or median ± standard deviation.

The median CEAP classification of all lower limbs was 3 (IQR, 0-4); the median Villalta score of all lower limbs was 7 (IQR, 0-10) for 34, 17, six, and 11 limbs with absent, mild, moderate, and severe symptoms, respectively, and the mean VCSS scores of all limbs were 4 ± 4.

### Technical parameters and feasibility test

#### Consistence test for hemodynamic parameters between 2D-PC MRI and 4D-Flow MRI

ICC was calculated to evaluate the hemodynamic parameters between 2D-PC MRI and 4D-Flow MRI. FR per cardiac cycle and Peak FV can be obtained for both 2D-PC MRI and 4D-Flow MRI, which are used to compare the consistency. For FR_IVC_ per cardiac cycle, FR_LCIV_ per cardiac cycle, FR_RCIV_ per cardiac cycle, FR_LCFV_ per cardiac cycle, and FR_RCFV_ per cardiac cycle, the ICC values are 0.851, 0.792, 0.946, 0.783, and 0.789 respectively. The ICC values for peak FV_IVC_, peak FV_LCIV_, peak FV_RCIV_, peak FV_LCFV_, and peak FV_RCFV_ were 0.936, 0.720, 0.965, 0.923, and 0.941, respectively. The results illustrated that there was consistence of hemodynamic parameters between 2D-PC and 4D-Flow MRI, which are shown in Supplementary Table II (online only).

#### Test of flow conservation for hemodynamic parameters

According to the principle of flow conservation, the sum of FR of the IVC should be equal to the total FR of the bilateral CIV. In this study, if the theoretically calculated value of IVC FR was *Q*_*I (calculate)*_, the measured values of IVC, LCIV, and RCIV FR were *Q*_*I*__*(measure)*_, *Q*_*L*__*(measure)*_ and *Q*_*R*__*(measure)*_, and *Q*_*I*__*(calculate)*_ *= Q*_*L*__*(measure)*_ *+ Q*_*R*__*(measure)*_, and *Q*_*I*__*(measure)*_ *= Q*_*I*__*(calculate)*_. However, because of the existence of measurement errors, the measured FR of IVC is usually not equal to the total measured FR of bilateral CIV, namely, *Q*_*I*__*(calculate)*_ *≠ Q*_*L*__*(calculate)*_ *+ Q*_*R*__*(calculate)*_ in numerical data. Therefore, to ensure that the measured hemodynamic parameters fulfilled the principle of flow conservation, theStudent *t*-test was used to evaluate the difference between *Q*_*I*__*(measure)*_ and *Q*_*I*__*(calculate)*_. The results shown in Supplementary Table III (online only) demonstrate that there was no statistically significant difference between *Q*_*I*__*(measure)*_ and *Q*_*I*__*(calculate)*_ (*P* > .05). Thus, the measured hemodynamic parameters complied with the principle of flow conservation. A schematic diagram (Supplementary Fig 2, online only) was used to illustrate mathematical relationships between the flow rates of the IVC, RCIV, and LCIV.

### Findings in patients with venous pathology

#### Differences of hemodynamic parameters between symptomatic and asymptomatic limbs

According on the presence of CVD symptoms, all lower limbs enrolled in this study were divided into two groups including symptomatic and asymptomatic limbs. Because the values of all parameters complied with normal distribution, the Student *t*-test was used to evaluate the difference in hemodynamic parameters between symptomatic and asymptomatic limbs. The results shown in Table II demonstrate that the flow rate-related parameters of CIV, peak FV, and pressure gradient of CFV measured by 2D-PC (FR per cardiac cycle:6.0 ± 3.1vs 8.5 ± 5.1; *P* = .01; FR per minutes: 0.4 ± 0.2 vs 0.7 ± 0.4; *P* = .01; Peak FV: 12.3 ± 6.5 vs 17.3 ± 8.6; *P* < .01) and 4D-Flow MRI (FR per cardiac cycle 6.9 ± 2.8 vs 8.7 ± 4.2; *P* = .04; Peak FV: 13.0 ± 5.5 vs 17.2 ± 7.1; *P* = .01; Pressure gradient: 0.1 ± 0.1 vs 0.2 ± 0.2; *P* = .01) were significantly different between the symptomatic and asymptomatic limbs.

**Table II:** Differences of hemodynamic parameters between symptomatic and asymptomatic limbs

Parameters	Symptomatic limbs (n = 42)	Asymptomatic limbs (n = 26)	*P* value
2D-PC MRI			
CIV			
FR per cardiac cycle	6.0 ± 3.1	8.5 ± 5.1	**.01**
FR per minutes	0.4 ± 0.2	0.7 ± 0.4	**.01**
Peak FV	17.5 ± 9.0	19.1 ± 7.3	.46
Minimum FV	1.1 ± 5.3	2.5 ± 4.2	.25
CFV			
FR per cardiac cycle	4.4 ± 2.5	4.9 ± 2.0	.33
FR per minutes	0.3 ± 0.2	0.4 ± 0.2	.28
Peak FV	12.3 ± 6.5	17.3 ± 8.6	**<.01**
Minimum FV_CFV_	1.7 ± 3.9	2.2 ± 4.6	.66
FR _CIV-CFV_ difference			
FR per cardiac cycle	1.6 ± 2.1	3.6 ± 4.3	**.01**
FR per minutes	0.1 ± 0.2	0.3 ± 0.4	**.04**
FV_CIV/CFV_ ratio			
Peak FV	5.2 ± 7.9	1.6 ± 8.7	**.07**
Minimum FV	-0.6 ± 5.1	0.4 ± 4.9	.44
4D-Flow MRI			
CIV			
FR per cardiac cycle	6.9 ± 2.8	8.7 ± 4.2	**.04**
Peak FV	19.5 ± 8.6	20.5 ± 9.1	.67
Pressure gradient_CIV_	0.2 ± 0.2	0.2 ± 0.1	.74
CFV			
FR per cardiac cycle	3.71 ± 1.91	3.88 ± 1.86	.72
Peak FV	13.0 ± 5.5	17.2 ± 7.1	**.01**
Pressure gradient	0.1 ± 0.1	0.2 ± 0.2	**.01**
FR _CIV-CFV_ difference			
FR per cardiac cycle	3.0 ± 2.8	4.8 ± 3.5	**.05**
FV_CIV/CFV_ ratio			
Peak FV	1.3 ± 0.6	1.5 ± 0.6	.44
Pressure gradient_CIV-CFV_ difference			
Pressure gradient	0.01 ± 0.2	0.04 ± 0.1	.44

*2D-PC MRI,* Two dimensional-phase contract magnetic resonance imaging; *4D-Flow MRI,* four-dimensional flow magnetic resonance imaging; *CFV,* common femoral vein; *CIV,* common iliac vein; *FR,* flow rate; *FV,* flow velocity.

Data are presented as median ± standard deviation.

Boldface *P* values indicate statistical significance.

#### Differences of hemodynamic parameters between limbs with and without iliac vein compression

According to the CTV images, all lower limbs enrolled in this study were divided into two groups: limbs with iliac vein compression and limbs without iliac vein compression. The Student *t*-test was used to evaluate the differences in hemodynamic parameters between limbs with and without iliac vein compression. The results illustrated that the flow rate-related parameters of CIV were statistically significantly different between the two groups (2P-PC MRI: FR per cardiac cycle: 5.3 ± 3.0 vs 7.6 ± 4.4; *P* = .03; FR difference: 1.3 ± 2.7 vs 2.8 ± 3.4) (4D-Flow MRI: FR per cardiac cycle: 6.1 ± 2.6 vs 8.2 ± 3.7; FR difference: 2.1 ± 3.7 vs 4.6 ± 3.1), which are listed in Table III.

**Table III:** Differences of hemodynamic parameters between limbs with and without iliac vein compression

Parameters	Limbs with iliac vein compression (n = 20)	Limbs without iliac vein compression (n = 48)	*P* value
2D-PC MRI			
CIV			
FR per cardiac cycle	5.3 ± 3.0	7.6 ± 4.4	**.03**
FR per minutes	0.4 ± 0.2	0.6 ± 0.4	**.04**
Peak FV	17.7 ± 10.3	18.3 ± 7.5	.80
Minimum FV	1.9 ± 6.0	1.6 ± 4.5	.79
CFV			
FR per cardiac cycle	4.0 ± 1.8	4.8 ± 2.4	.20
FR per minutes	0.3 ± 0.1	0.4 ± 0.2	.22
Peak FV	13.9 ± 6.9	14.5 ± 8.2	.79
Minimum FV	2.8 ± 4.5	1.5 ± 4.0	.23
FR _CIV-CFV_ difference			
FR per cardiac cycle	1.3 ± 2.7	2.8 ± 3.4	**.04**
FR per minutes	0.1 ± 0.2	0.2 ± 0.3	**.04**
FV_CIV/CFV_ ratio			
Peak FV	3.8 ± 9.3	3.8 ± 8.0	.98
Minimum FV	-0.9 ± 5.5	0.03 ± 4.8	.47
4D-Flow MRI			
CIV			
FR per cardiac cycle	6.1 ± 2.6	8.2 ± 3.7	**<.01**
Peak FV	18.7 ± 8.9	20.4 ± 8.7	.46
Pressure gradient	0.2 ± 0.1	0.2 ± 0.3	.96
CFV			
FR per cardiac cycle	3.4 ± 1.6	3.9 ± 2.0	.21
Peak FV	13.6 ± 5.4	15.0 ± 6.8	.37
Pressure gradient	0.1 ± 0.1	0.1 ± 0.1	.31
FR _CIV-CFV_ difference			
FR per cardiac cycle	2.11 ± 3.72	4.56 ± 3.06	**.04**
FV_CIV/CFV_ ratio			
Peak FV	1.3 ± 0.4	1.4 ± 0.7	.60
Pressure gradient_CIV-CFV_ difference			
Pressure gradient	0.01 ± 0.1	0.03 ± 0.2	.61

*2D-PC MRI,* Two dimensional-phase contract magnetic resonance imaging; *4D-Flow MRI,* four-dimensional flow magnetic resonance imaging; *CFV,* common femoral vein; *CIV,* common iliac vein; *FR,* flow rate; *FV,* flow velocity.

Data are presented as median ± standard deviation.

Boldface *P* values indicate statistical significance.

#### Relationship between hemodynamic and anatomic parameters

The results listed in Supplementary Table IV (online only) illustrated that there was a potential negative correlation between the FR per minute of CIV measured by 2D-PC MRI and the area stenosis (*P* = .06; r = −0.7). Furthermore, peak and minimum FV (CIV/CFV) ratio were correlated negatively with the confluence angle of CIV (*P* < .01; r = −0.5; *P* = .03; r = −0.3).

#### Relationship between hemodynamic parameters and severity of symptoms in all limbs

A total of 68 lower limbs, including 42 symptomatic limbs and 26 asymptomatic limbs, were enrolled in this correlation analysis. Each limb was evaluated by CEAP classification, Villalta, and VCSS scores according to the severity of symptoms, with all asymptomatic limbs treated as Lv.0, 0 score, and 0 score in CEAP classification, Villalta, and VCSS scores. The results listed in Table IV illustrate that for 2D-PC MRI, the flow rate-related parameters of CIV, such as FR per cardiac cycle (*P* < .01; r = −0.3) and minute (*P* < .01; r = −0.3) and FR (CIV−CFV) difference (*P* = .03; r = −0.3) were correlated negatively with the severity of symptoms. Furthermore, there were positive and negative relationships between the severity of symptoms and the FV (CIV/CFV) ratio (*P* < .0; r = 0.3) and peak FV of CFV (*P* < .01; r = −0.4), respectively. These phenomena were also observed in the parameters measured by 4D-Flow MRI as well.

**Table IV:** Relationship between hemodynamic parameters and severity of symptom in all limbs

CEAP			Villalta			VCSS		
Parameters	Correlation Index (r)	*P* value	Parameters	Correlation Index (r)	*P* value	Parameters	Correlation Index (r)	*P* value
2D-PC MRI								
CIV								
FR per cardiac cycle	−0.3	**<.01**	FR per cardiac cycle	−0.3	**.01**	FR per cardiac cycle	−0.3	**.02**
FR per minute	−0.3	**<.01**	FR per minute	−0.3	**.01**	FR per minute	−0.3	**.02**
Peak FV	−0.2	**.08**	Peak FV	−0.1	.48	Peak FV	−0.1	.29
Minimum FV	0.1	.32	Minimum FV	<−0.1	.96	Minimum FV	<−0.1	.88
CFV								
FR per cardiac cycle	−0.2	**.07**	FR per cardiac cycle	−0.2	**.08**	FR per cardiac cycle	−0.2	**.06**
FR per minute	−0.2	**.06**	FR per minute	−0.2	**.08**	FR per minute	−0.2	**.04**
Peak FV	−0.4	**<.01**	Peak FV	−0.4	**<.01**	Peak FV	−0.4	**<.01**
Minimum FV	−0.1	.29	Minimum FV	0.1	.41	Minimum FV	0.1	.66
FR (CIV−CFV) difference								
FR per cardiac cycle	−0.3	**.03**	FR per cardiac cycle	−0.2	**.09**	FR per cardiac cycle	−0.2	**.06**
FR per minute	−0.2	**.04**	FR per minute	−0.2	**.08**	FR per minute	−0.2	**.06**
FV (CIV/CFV) ratio								
Peak FV	0.3	**<.01**	Peak FV	0.3	**.02**	Peak FV	0.3	**.02**
Minimum FV	0.1	.33	Minimum FV	<−0.1	.71	Minimum FV	<0.1	.98
4D-Flow MRI								
CIV								
FR per cardiac cycle	−0.2	**.07**	FR per cardiac cycle	−0.2	**.05**	FR per cardiac cycle	−0.2	**.05**
Peak FV	−0.1	.26	Peak FV	−0.1	.60	Peak FV	−0.1	.40
Pressure gradient	−0.2	**.06**	Pressure gradient	<−0.1	.98	Pressure gradient	<−0.1	.70
CFV								
FR per cardiac cycle	−0.1	.38	FR per cardiac cycle	<−0.1	.82	FR per cardiac cycle	−0.1	.42
Peak FV	−0.4	**.01**	Peak FV	−0.4	**.01**	Peak FV	−0.4	**.01**
Pressure gradient	−0.4	**.01**	Pressure gradient	−0.4	**.01**	Pressure gradient	−0.4	**.01**
FR, pressure gradient (CIV−CFV) difference and FV (CIV/CFV) ratio								
FR per cardiac cycle	−0.2	.15	FR per cardiac cycle	−0.2	**.09**	FR per cardiac cycle	−0.2	**.07**
Peak FV	0.3	**.02**	Peak FV	0.3	**.02**	Peak FV	0.3	**.02**
Pressure gradient	0.2	.18	Pressure gradient	0.2	**.08**	Pressure gradient	0.2	.12

*2D-PC MRI,* Two dimensional-phase contract magnetic resonance imaging; *4D-Flow MRI,* four-dimensional flow magnetic resonance imaging; *CEAP,* clinical-etiological-anatomical-pathophysiological; *CFV,* Common femoral vein; *CIV,* common iliac vein; *FR,* flow rate; *FV,* flow velocity; *VCSS,* venous clinical severity score.

Data are presented as median ± standard deviation.

Boldface *P* values indicate statistical significance.

#### Relationship between hemodynamic parameters and severity of symptoms in lower limbs with iliac vein compression

Twenty limbs with iliac vein compression were included in the correlation analysis. Due to the absence of asymptomatic limbs with iliac vein compression in this study, all the asymptomatic limbs were excluded from this correlation analysis. The results listed in Table V illustrate that, for 2D-PC MRI, there was a potential correlation between the severity of symptoms and the flow rate-related parameters of CIV and CFV, such as FR per cardiac cycle (CIV: *P* = .07; r = −0.4; CFV: *P* = .02; r = −0.5) and minute (CIV: *P* = .04; r = −0.4; CFV: *P* = .07; r = −0.5). However, these phenomena, especially the correlation between symptom severity and flow rate-related parameters of CIV, were not observed in 4D-Flow MRI.

**Table V:** Relationship between hemodynamic parameters and severity of symptom in lower limbs with iliac vein compression

CEAP			Villalta			VCSS		
Parameters	Correlation index (r)	*P* value	Parameters	Correlation index (r)	*P* value	Parameters	Correlation index (r)	*P* value
2D-PC MRI								
CIV								
FR per cardiac cycle	−0.3	.22	FR per cardiac cycle	−0.4	**.07**	FR per cardiac cycle	−0.3	.17
FR per minute	−0.3	.13	FR per minute	−0.4	**.04**	FR per minute	−0.3	.23
Peak FV	−0.1	.73	Peak FV	0.3	.89	Peak FV	<0.1	.98
Minimum FV	0.3	.17	Minimum FV	0.2	.31	Minimum FV	0.4	**.06**
CFV								
FR per cardiac cycle	−0.5	**.02**	FR per cardiac cycle	−0.7	**<.01**	FR per cardiac cycle	−0.4	**.05**
FR per minute	−0.5	**.01**	FR per minute	−0.6	**<.01**	FR per minute	−0.4	**.07**
Peak FV	−0.4	.12	Peak FV	−0.3	.26	Peak FV	−0.2	.41
Minimum FV	−0.3	.28	Minimum FV	−0.2	.32	Minimum FV	−0.1	.73
FR (CIV−CFV) difference								
FR per cardiac cycle	−0.2	.62	FR per cardiac cycle	−0.1	.77	FR per cardiac cycle	<−0.1	.92
FR per minute	−0.2	.62	FR per minute	−0.1	.79	FR per minute	<−0.1	.93
FV (CIV/CFV) ratio								
Peak FV	<0.1	.94	Peak FV	−0.2	.68	Peak FV	−0.1	.86
Minimum FV	0.3	.46	Minimum FV	0.2	.57	Minimum FV	0.3	.42
4D-Flow MRI								
CIV								
FR per cardiac cycle	<−0.1	.89	FR per cardiac cycle	−0.2	.51	FR per cardiac cycle	−0.2	.38
Peak FV	0.1	.62	Peak FV	0.1	.71	Peak FV	−0.1	.75
Pressure gradient	0.2	.49	Pressure gradient	0.2	.38	Pressure gradient	0.2	.49
CFV								
FR per cardiac cycle	−0.4	**.05**	FR per cardiac cycle	−0.4	**.07**	FR per cardiac cycle	−0.5	**.04**
Peak FV	−0.5	**.04**	Peak FV	−0.5	**.04**	Peak FV	−0.4	.11
Pressure gradient	−0.3	.19	Pressure gradient	−0.3	.22	Pressure gradient	−0.3	.24
FR, pressure gradient (CIV−CFV) difference and FV (CIV/CFV) ratio								
FR per cardiac cycle	0.3	.25	FR per cardiac cycle	0.1	.73	FR per cardiac cycle	0.1	.78
Peak FV	0.4	.06	Peak FV	0.5	**.02**	Peak FV	0.3	.21
Pressure gradient	0.3	.22	Pressure gradient	0.3	.20	Pressure gradient	0.3	.29

*2D-PC MRI,* Two dimensional-phase contract magnetic resonance imaging; *4D-Flow MRI,* four-dimensional flow magnetic resonance imaging; *CEAP,* clinical-etiological-anatomical-pathophysiological; *CFV,* common femoral vein; *CIV,* common iliac vein; *FR,* flow rate; *FV,* flow velocity; *VCSS,* venous clinical severity score.

Data are presented as median ± standard deviation.

Boldface *P* values indicate statistical significance.

## Discussion

On the basis of our analysis, we found that, compared with asymptomatic limbs or limbs without iliac vein compression, the values of CIV FR-related parameters in symptomatic limbs and limbs with iliac vein compression, including FR per cardiac cycle, FR per minute, and FR (CIV−CFV) difference acquired by 2D-PC MRI and 4D-Flow MRI, were significantly lower. These results were similar with that reported in a previous study by Labropoulos et al^18^ as well, who found that the flow volume measured by Doppler ultrasound in the CIV of asymptomatic limbs was significantly greater than that of symptomatic limbs. As for patients with CVD, the descent of lower limb vein backflow volume, caused by iliac vein compression, vein valve disorder, etc, was responsible for venous hypertension, indicating lower limb symptoms such as swelling, hyperpigmentation, and vein ulcer. Furthermore, iliac vein compression resulted in hemodynamic changes and redistribution, and a previous study illustrated that in some IVCS cases, due to hypertension in the veins of the lower limbs, collateral vessels formed and could be observed by CTV or digital subtraction angiography^19^^,^^20^^,^^21^ draining the blood in the congestive limbs to the contralateral limbs or proximal vein, which caused a decrease in the FR of CIV and FR difference between CIV and CFV. Therefore, the changes in FR could be treated as indicators to identify CVD as well. Our results verified their findings.

In the present study, we found that some hemodynamic parameters related to the severity of lower limb symptoms in patients enrolled in this study. The FR of CIV, CFV, and the FR difference between CIV and CFV were correlated with the severity rank of symptoms in CVD. And the FR of the CFV were correlated with the severity rank of symptoms in limbs with iliac vein compression, with a potential correlation between the FR of the CIV and severity rank of symptoms in limbs with iliac vein compression. A higher symptom score represented a worse venous hypertension in symptomatic limbs, which was caused by a decrease in venous backflow, manifesting as a descent of FR in hemodynamic parameters. These phenomena were also reported by Chongqing Medical University in a prospective study,^22^ which illustrated that the FR descent of the CIV and changes in FR differences between the CIV and EIV correlated significantly with lower limb symptom severity. However, in our study, we did not find that the FR difference between CIV and CFV correlated with the severity of symptoms in limbs with iliac vein compression. This may be due to the existence of varicose veins in some patients with iliac vein compression in our cohort, which can also affect the severity of CVD symptoms.

In the present study, we found that FV-related parameters, such as peak FV in CFV, were negatively correlated with the severity of symptoms in CVD. Although there was no statistically significant difference between symptomatic and asymptomatic limb FV in our study, the mean values of CIV and CFV peak FV of symptomatic limbs and limbs with iliac vein compression were slightly higher than those of asymptomatic limbs and limbs without iliac vein compression, respectively. This trend was similar to a previous case-control study indicating that a peak FV ≤10 cm/s was 92.1% (95% confidence interval, 79.2%-97.3%) sensitive to detect venous stent obstruction, whereas venous stent obstruction was ruled out if the peak FV was more than 10 cm/s.^23^ Because of the diversity of vessel diameter in every individual, the interaction between FR, FV, and diameter according to Bernoulli’s principle, it should be emphasized that the descent of venous backflow volume was the most important cause for the appearance of lower limb symptoms. Namely, FR was a more reliable parameter to evaluate the symptoms than FV, although the congestion and swelling of the lower limbs indeed caused the descent of FV.

The consistency and differences between the parameters of 2D-PC MRI and 4D-Flow were also evaluated in this study. Compared with 2D-PC MRI, 4D-Flow MRI is an attractive method, as it is better at evaluating complex multidirectional hemodynamic flow patterns because it can capture velocity data in all three directions.^24^ Our result illustrated that there was consistency between the value of hemodynamic parameters of 2D-PC MRI and 4D-Flow MRI, which was certified by previous studies as well.^17^^,^^25^^,^^26^ Furthermore, the mean values of FR measured by 4D-Flow MRI were higher than those measured by 2D-PC MRI, as 4D-Flow MRI measured velocity in all spatial directions and has superior spatial coverage to 2D-PC MRI, similar to studies by Nordmeyer et al and Bollache et al.^25^^,^^27^ However, in the present study, compared with 4D-Flow MRI, the correlation between parameters of 2D-PC MRI and the severity of symptoms in limbs with iliac vein compression was more significant. The potential reason for this was the difference in measurement, data post processing, and resolution ratios of images between 2D-PC and 4D-Flow MRI. First, for 2D-PC MRI, the step of selecting measurement sites was in MRI scanning period, whereas for the 4D-Flow MRI, the measurement point is determined during data post processing, which would cause the potential biases of measurement sites between 2D-PC and 4D-Flow MRI. In addition, the resolution ratio of phase and amplitude image in 2D-PC MRI was greater than 4D-Flow MRI. Furthermore, in the data post processing period of 4D-Flow MRI, due to the anatomical relationship between the common iliac artery (CIA) and CIV, compared with CIV, the flow signal of CIA in 4D-Flow was identified and captured by the software preferentially, which would cause personal error when the ROI of CIV was determined as the flow signal of CIV was disturbed by the adjacent iliac artery.^28^ Therefore, due to the existence of measurement errors, the relationship between parameters and severity of symptoms was not significant when a small sample size (n = 20) was included in the analysis. If the sample size increased (n = 68), potential measurement error was eliminated. Therefore, because of the reasons for resolution ratio of image and data-post processing, for patients with CVD, the hemodynamic parameters measured by 2D-PC MRI were more accurate than 4D-Flow MRI. However, 4D-Flow MRI possessed its distinct advantage as it can obtain a streamlined chart to evaluate the more complex flow pattern more visually.

This study had some limitations. First, the sample size of this study was small and from a single center. Whether these results could be replicated in other centers needs further research. Second, all hemodynamic parameters were acquired in the supine position, but patients were often in an upright position in the natural state. Therefore, the change of body position may influence the value of hemodynamic parameters. In addition, hemodynamic parameters can be influenced by many other factors, such as the effects of respiratory rate, posture, and exercise, which were not analyzed in this study. Furthermore, iliac vein compression was identified by CTV, which could cause that some patients could been miscategorized potentially, as CTV was not as sensitive as the combination of venography and intravascular ultrasound. So more detailed iliac vein hemodynamic investigation is warranted in the future. Additionally, this research was designed as an exploratory study and did not include follow-up data regarding whether patients eventually underwent stenting or other treatments. Therefore, further research will be performed to investigate the relationship between diagnostic findings and subsequent treatment outcomes, such as stenting success, which would be highly valuable to the clinical practice.

## Conclusions

CVD causes hemodynamic changes in the lower limb veins. The decrease in FR-related hemodynamic parameters measured by 2D-PC and 4D-Flow MRI in CIV have the potential to identify CVD or iliac vein compression and classify the severity of lower limb symptoms. Furthermore, hemodynamic parameters measured by 2D-PC and 4D-Flow MRI not only complied with flow rate conservation but also maintained consistency. In conclusion, owing to the advantages of no contrast enhancement and visualization flow field, 2D-PC and 4D-Flow MRI possess the potential clinical value of assisting CVD and iliac vein compression diagnosis and lower limb symptom classification.

## Author contributions

Conception and design: TX, MZ, SF, ZS

Analysis and interpretation: TX, XS, YD

Data collection: TX, YS, XL, ZZ, CY

Writing the article: TX, YD, XL, ZZ

Critical revision of the article: XS, YS, MZ, SF, CY, ZS

Final approval of the article: TX, XS, YS, MZ, YD, XL, ZZ, SF, CY, ZS

Statistical analysis: XS, YD, XL

Obtained funding: ZS

Overall responsibility: ZS

TX, XS, and YS contributed equally to this article and share co-first authorship.

## Funding

This work was sponsored by 10.13039/501100001809National Natural Science Foundation of China (81870342).

## Disclosures

None.

## References

[1] Costa D., Andreucci N., Ielapi G. (2023). Molecular determinants of chronic venous disease: a Comprehensive Review. Int J Mol Sci.

[2] Poyyamoli S., Mehta P., Cherian M. (2021). May-Thurner syndrome. Cardiovasc Diagn Ther.

[3] Radaideh Q., Patel N.M., Shammas N.W. (2019). Iliac vein compression: epidemiology, diagnosis and treatment. Vasc Health Risk Manag.

[4] Kibbe M.R., Ujiki M., Goodwin A.L., Eskandari M., Yao J., Matsumura J. (2004). Iliac vein compression in an asymptomatic patient population. J Vasc Surg.

[5] Knuttinen M.G., Naidu S., Oklu R. (2017). May-Thurner: diagnosis and endovascular management. Cardiovasc Diagn Ther.

[6] Assi I.Z., Lynch S.R., Samulak K. (2023). An ultrasound imaging and computational fluid dynamics protocol to assess hemodynamics in iliac vein compression syndrome. J Vasc Surg Venous Lymphat Disord.

[7] Lorenção de Almeida B., Rossi F.H., Guerra de Moraes Rego Sousa A. (2018). Correlation between venous pressure gradients and intravascular ultrasound in the diagnosis of iliac vein compression syndrome. J Vasc Surg Venous Lymphat Disord.

[8] Hurst D.R., Forauer A.R., Bloom J.R. (2001). Diagnosis and endovascular treatment of iliocaval compression syndrome. J Vasc Surg.

[9] Wentland A.L., Grist T.M., Wieben O. (2013). Repeatability and internal consistency of abdominal 2D and 4D phase contrast MR flow measurements. Acad Radiol.

[10] Adriaans B.P., Westenberg J.J.M., van Cauteren Y.J.M. (2020). Clinical assessment of aortic valve stenosis: Comparison between 4D flow MRI and transthoracic echocardiography. J Magn Reson Imaging.

[11] Zhang G.L., Zhou Y.R., Wu D. (2022). Overwise of 4D Flow MRI hemodynamic imaging and its clinical practise. Radio Practise.

[12] Wåhlin A., Eklund A., Malm J. (2022). 4D flow MRI hemodynamic biomarkers for cerebrovascular diseases. J Intern Med.

[13] Hyodo R., Takehara Y., Naganawa S. (2022). 4D Flow MRI in the portal venous system: imaging and analysis methods, and clinical applications. Radiol Med.

[14] Burris N.S., Hope M.D. (2015). 4D flow MRI applications for aortic disease. Magn Reson Imaging Clin N Am.

[15] Wang H., Jia W., Xi Y. (2024). Morphometric and hemodynamic analysis of the compressed iliac vein. J Endovasc Ther.

[16] Phlebology Group (2022). Vascular surgeon branch, Chinese Medical Doctor Association, guidelines for the diagnosis and treatment of common venous diseases (2022 Edition). Chin J Vasc Surg.

[17] Hautanen S., Kiljander T., Korpela T. (2023). 4D flow versus 2D phase contrast MRI in populations with Bi- and Tricuspid aortic valves. In Vivo.

[18] Labropoulos N., Borge M., Pierce K., Pappas P.J. (2007). Criteria for defining significant central vein stenosis with duplex ultrasound. J Vasc Surg.

[19] Xu Y., Wu J., Cheng Y. (2023). Evaluation of 3-dimensional rotational venography for the diagnosis of non-thrombotic iliac venous lesion. Front Cardiovasc Med.

[20] Thomas M.L., Fletcher E.W., Cockett F.B., Negus D. (1967). Venous collaterals in external and common iliac vein obstruction. Clin Radiol.

[21] Neglén P., Raju S. (1993). Detection of outflow obstruction in chronic venous insufficiency. J Vasc Surg.

[22] Chen Z.H., Huang Y., Wang L.P., Peng M.Y., Li C., Huang W. (2022). Preliminary study of hemodynamics of iliac venous compression syndrome using magnetic resonance imaging. J Vasc Surg Venous Lymphat Disord.

[23] Sebastian T., Barco S., Engelberger R.P. (2020). Duplex ultrasound investigation for the detection of obstructed iliocaval venous stents. Eur J Vasc Endovasc Surg.

[24] Lotz J., Meier C., Leppert A., Galanski M. (2002). Cardiovascular flow measurement with phase-contrast MR imaging: basic facts and implementation. Radiographics.

[25] Bollache E., Ooij van P., Powell A. (2016). Comparison of 4D flow and 2D velocity-encoded phase contrast MRI sequences for the evaluation of aortic hemodynamics. Int J Cardiovasc Imaging.

[26] Secchi F., Monti C.B., Capra D. (2021). Carotid phase-contrast magnetic resonance before treatment: 4D-flow versus standard 2D imaging. Tomography.

[27] Nordmeyer S., Riesenkampff E., Messroghli D. (2013). Four-dimensional velocity-encoded magnetic resonance imaging improves blood flow quantification in patients with complex accelerated flow. J Magn Reson Imaging.

[28] Bissell M.M., Raimondi F., Ait Ali L. (2023). 4D Flow cardiovascular magnetic resonance consensus statement: 2023 update. J Cardiovasc Magn Reson.

