# Supplementary material for: Evaluation of hemodynamic characteristics of iliac vein in chronic venous disease and iliac vein compression syndrome patients using magnetic resonance image: A prospective study
Source: J Vasc Surg Venous Lymphat Disord. 2025 Apr 8;13(5):102247. doi: 10.1016/j.jvsv.2025.102247 (PMC12158526; doi:10.1016/j.jvsv.2025.102247)
Supplement: Supplementary Material [file mmc1.docx]

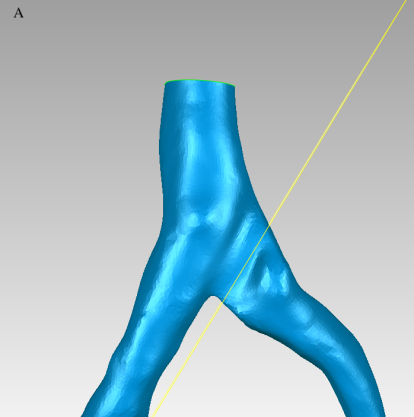

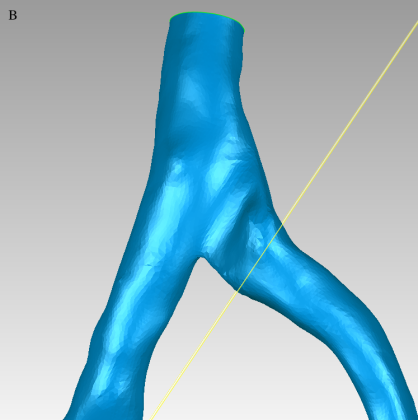

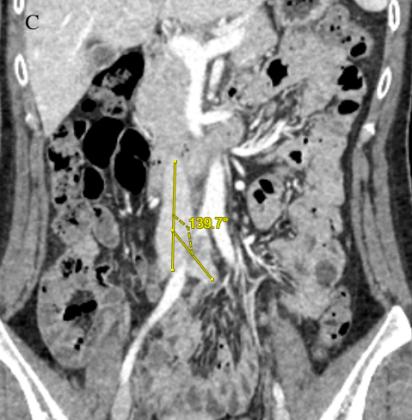


Supplementary Figure S1 The methods of area stenosis (A, B) and confluence of angle measurement (C) . A was the cross-section of compression site. B was the cross-section of distal normal vessel. C reflects the confluence of angle between the CIV and the IVC. The angle marked in the image is 139.7°, and the supplementary angle of 40.3° is used to describe the confluence of angle.

Supplementary Table S1 The hemodynamic parameters enrolled in this study

| Parameter | Definition | Unit | Measurement Method |
| --- | --- | --- | --- |
| Flow rate (FR) per cardiac cycle | The total volume of blood passing through the cross-section of target vessel during one complete cardiac cycle | mL/cardiac cycle | 2D PC-MRI  4D-Flow MRI |
| Flow rate (FR) per minutes | The total volume of blood passing through the cross-section of target vessel per minute | L/min | 2D PC-MRI |
| Peak flow velocity (FV ) | The highest blood flow velocity recorded within the target vessel during a cardiac cycle | cm/s | 2D PC-MRI  4D-Flow MRI |
| Minimum flow velocity (FV ) | The lowest blood flow velocity recorded within the target vessel during a cardiac cycle | cm/s | 2D PC-MRI |
| Pressure gradient | The difference in venous blood pressure between two anatomical points | mmHg | 4D-Flow MRI |

Supplementary Table S2 Consistence test for hemodynamic parameters between 2D-PC MRI and 4D-Flow MRI

| Parameters | 2D-PC MRI (n=34) | 4D-Flow MRI (n=34) | ICC value | P value |
| --- | --- | --- | --- | --- |
| **IVC** |  |  |  |  |
| FR per cardiac cycle (mL/cardiac cycle) | 14.8±4.8 | 15.7±4.1 | 0.851 | **<0.01** |
| Peak FV (cm/s) | 23.2±9.5 | 25.3±9.6 | 0.936 | **<0.01** |
| **LCIV** |  |  |  |  |
| FR per cardiac cycle (mL/cardiac cycle) | 5.9±3.0 | 7.0±3.0 | 0.792 | **<0.01** |
| Peak FV (cm/s) | 15.3±5.7 | 17.6±7.2 | 0.720 | **<0.01** |
| **RCIV** |  |  |  |  |
| FR per cardiac cycle (mL/cardiac cycle) | 7.9±4.9 | 8.2±3.9 | 0.946 | **<0.01** |
| Peak FV (cm/s) | 21.0±9.6 | 22.2±9.6 | 0.965 | **<0.01** |
| **LCFV** |  |  |  |  |
| FR per cardiac cycle (mL/cardiac cycle) | 4.4±2.3 | 3.8±1.8 | 0.783 | **<0.01** |
| Peak FV (cm/s) | 13.3±6.6 | 13.9±5.3 | 0.923 | **<0.01** |
| **RCFV** |  |  |  |  |
| FR per cardiac cycle (mL/cardiac cycle) | 3.7±2.0 | 4.7±2.3 | 0.789 | **<0.01** |
| Peak FV (cm/s) | 15.3±8.8 | 15.3±7.8 | 0.941 | **<0.01** |

Supplementary Table S3 Test of flow conservation for hemodynamic parameters

| Parameters | *Q_I (measure)_*(n=34) | *Q_I (calculate)_*(n=34) | P value |
| --- | --- | --- | --- |
| **2D-PC MRI** |  |  |  |
| FR per cardio cycle (mL/cardiac cycle) | 14.8±5.9 | 14.1±5.7 | 0.59 |
| FR per minutes (L/min) | 1.1±0.5 | 1.1±0.5 | 0.50 |
| **4D-Flow MRI** |  |  |  |
| FR per cardio cycle (mL/cardiac cycle) | 15.7±4.1 | 15.2±4.1 | 0.64 |


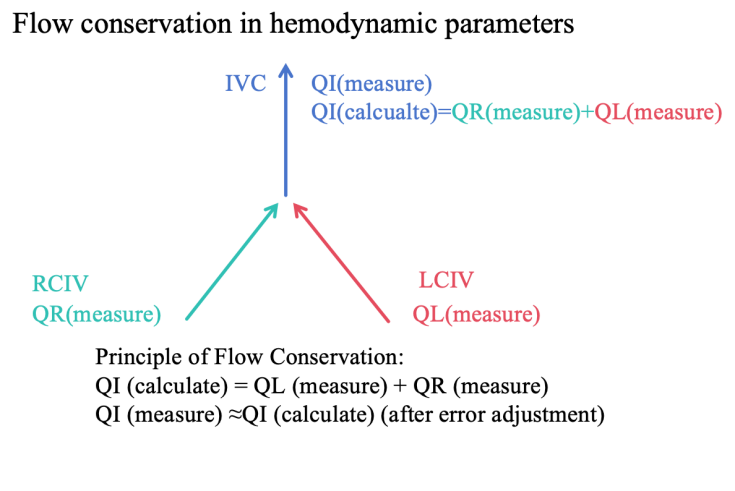


Supplementary Figure S2 Flow conservation of hemnodynamic parameters

Supplementary Table S4 Relationship between hemodynamic and anatomic parameters

| Area Stenosis | | | Confluence angle of CIV | | |
| --- | --- | --- | --- | --- | --- |
| Parameters | Correlation  Index (r) | P  value | Parameters | Correlation  Index (r) | P  value |
| **2D-PC MRI** |  |  |  |  |  |
| **CIV** |  |  |  |  |  |
| FR per cardiac cycle | -0.5 | 0.24 | FR per cardiac cycle | -0.1 | 0.62 |
| **FR per minute** | **-0.7** | **0.06** | FR per minute | <-0.1 | 0.96 |
| Peak FV | -0.1 | 0.75 | Peak FV | -0.2 | 0.14 |
| Minimum FV | <0.1 | 0.93 | Minimum FV | -0.1 | 0.75 |
| **CFV** |  |  |  |  |  |
| FR per cardiac cycle | -0.4 | 0.28 | FR per cardiac cycle | 0.1 | 0.47 |
| FR per minute | -0.4 | 0.31 | FR per minute | 0.1 | 0.43 |
| Peak FV | -0.4 | 0.35 | Peak FV | 0.1 | 0.34 |
| Minimum FV | 0.1 | 0.85 | Minimum FV | 0.3 | 0.08 |
| **FR(CIV-CFV) Difference** | | | | | |
| FR per cardiac cycle | -0.2 | 0.59 | FR per cardiac cycle | -0.2 | 0.12 |
| FR per minute | -0.4 | 0.29 | FR per minute | -0.2 | 0.31 |
| **FV(CIV/CFV) Ratio** | | | | | |
| Peak FV | -0.1 | 0.80 | **Peak FV** | **-0.5** | **<0.01** |
| Minimum FV | -0.1 | 0.78 | **Minimum FV** | **-0.3** | **0.03** |
| **4D-Flow MRI** |  |  |  |  |  |
| **CIV** |  |  |  |  |  |
| FR per cardiac cycle | -0.4 | 0.37 | FR per cardiac cycle | -0.1 | 0.65 |
| Peak FV | -0.1 | 0.78 | Peak FV | -0.1 | 0.76 |
| Pressure gradient | -0.6 | 0.19 | Pressure gradient | -0.2 | 0.27 |
| **CFV** |  |  |  |  |  |
| FR per cardiac cycle | -0.4 | 0.36 | FR per cardiac cycle | -0.2 | 0.27 |
| Peak FV | -0.6 | 0.20 | Peak FV | <0.1 | 0.97 |
| Pressure gradient | -0.5 | 0.30 | Pressure gradient | -0.2 | 0.32 |
| **FR, Pressure gradient (CIV-CFV) Difference & FV(CIV/CFV) Ratio** | | | | | |
| FR per cardiac cycle | -0.1 | 0.80 | FR per cardiac cycle | 0.1 | 0.72 |
| Peak FV | -0.1 | 0.82 | Peak FV | 0.1 | 0.50 |
| Pressure gradient | 0.1 | 0.84 | Pressure gradient | -0.1 | 0.47 |
